# A genome-wide scan for genes under balancing selection in *Drosophila melanogaster*

**DOI:** 10.1186/s12862-016-0857-z

**Published:** 2017-01-13

**Authors:** Myriam Croze, Andreas Wollstein, Vedran Božičević, Daniel Živković, Wolfgang Stephan, Stephan Hutter

**Affiliations:** 1 Section of Evolutionary Biology, Department of Biology II, University of Munich (LMU), Grosshaderner Str. 2, 82152 Planegg-Martinsried, Germany; 2 Center of Food and Life Sciences Weihenstephan, Technische Universität München, 85354 Freising, Germany; 3 Natural History Museum Berlin, 10115 Berlin, Germany

**Keywords:** Balancing selection, Genome scan, *Drosophila melanogaster*, Population genetics

## Abstract

**Background:**

In the history of population genetics balancing selection has been considered as an important evolutionary force, yet until today little is known about its abundance and its effect on patterns of genetic diversity. Several well-known examples of balancing selection have been reported from humans, mice, plants, and parasites. However, only very few systematic studies have been carried out to detect genes under balancing selection. We performed a genome scan in *Drosophila melanogaster* to find signatures of balancing selection in a derived (European) and an ancestral (African) population. We screened a total of 34 genomes searching for regions of high genetic diversity and an excess of SNPs with intermediate frequency.

**Results:**

In total, we found 183 candidate genes: 141 in the European population and 45 in the African one, with only three genes shared between both populations. Most differences between both populations were observed on the X chromosome, though this might be partly due to false positives. Functionally, we find an overrepresentation of genes involved in neuronal development and circadian rhythm. Furthermore, some of the top genes we identified are involved in innate immunity.

**Conclusion:**

Our results revealed evidence of genes under balancing selection in European and African populations. More candidate genes have been found in the European population. They are involved in several different functions.

**Electronic supplementary material:**

The online version of this article (doi:10.1186/s12862-016-0857-z) contains supplementary material, which is available to authorized users.

## Background

In most species, a high level of genetic polymorphism has been observed, and the causes of this high diversity have been widely debated. Dobzhansky proposed the ‘balanced hypothesis’, which suggests that many genes are polymorphic and that these polymorphisms are maintained by heterozygote advantage [1]. However, systematic studies that have been carried out on various organisms have so far reported little evidence of balancing selection [2–8].

Balancing selection is characterized by an increased genetic diversity because it maintains alleles at intermediate frequencies within populations. It involves various mechanisms: besides heterozygote advantage (also called overdominant selection [9, 10]) frequency-dependent selection [11] and local adaptation in substructured populations [12] have been proposed.

One famous case of heterozygote advantage is the sickle cell hemoglobin polymorphism in humans, which is maintained in environments in which *Plasmodium falciparum* is endemic [13]. Furthermore, some antigen genes are under overdominant selection in *P. falciparum* [7]. Several examples of negative frequency-dependent selection are also known, as for instance in the Major Histocompatibility Complex (MHC) in vertebrates [14–18] and in resistance genes (R-genes) in plants [19–21]. Frequency-dependent selection may be linked to coevolution between hosts and pathogens and, more specifically, to the trench warfare scenario in which polymorphisms in the host and the pathogen may be maintained for a long time [21, 22]. In *Drosophila*, it has been suggested that immune genes might be evolving under this type of balancing selection due to host-parasite interactions [23–25].

Most of the examples of balancing selection have been found in humans [4, 26–28], bacteria [5, 7], mice [29] and plants [30–32], and to a lesser extent in *D. melanogaster* (but see [33–37]). Although limited in number, these examples seem to suggest that, in addition to immunity genes, genes under balancing selection are also involved in other functions, such as the ABO blood group in primates [38] or the S-locus (determining self-incompatibility) in plants [39].

The reason why our understanding of balancing selection is limited to relatively few loci may be mainly due to the difficulty associated with detecting this type of selection at the whole-genome level. However, with the new technologies to analyze large datasets and the availability of whole-genome sequence data with high densities of polymorphisms, it should be possible to identify signatures of balancing selection if they exist. Only a few of these examples were detected by systematic searches (i.e. whole genome scans). An exception presents the work by Andrés et al. [4] who analyzed 13,400 human genes using methods based on the HKA test [40] and the site-frequency-spectrum (SFS). They found 60 candidates genes under balancing selection in two human populations. Several Drosophila studies have also recently obtained encouraging results in this respect [8, 36, 37]. Based on whole-genome sequence data, it has been shown that some genes share non-synonymous polymorphisms between *D. melanogaster* and *D. simulans* [34]. Such trans-species polymorphisms are expected to occur in the case of ancient balancing selection [41, 42]. Furthermore, Comeron [35] used a background selection model to look for the spatial distribution of polymorphisms and substitutions around selective sites as well as the allele frequencies surrounding polymorphic sites. He also found some new candidate genes for balancing selection in *D. melanogaster*. Interestingly, these genes are not only related to immunity, but are involved in an array of biological processes such as sensory perception of chemical stimuli, olfactory behavior, and inter-male aggressive behavior. However, more analyses are needed to increase our knowledge about balancing selection.

In this study, we performed a systematic genome-wide scan to search for signatures of balancing selection in *D. melanogaster*. Following our preliminary work [8], we used next-generation-sequencing (NGS) data to identify targets of balancing selection in a derived (European) and an ancestral (African) population. We searched for evidence of balancing selection based on two criteria: high levels of polymorphism compared to neutral expectations and a distortion of the SFS toward intermediate frequencies. To ensure significance of our results we performed coalescent simulations using appropriate demographic models.

## Methods

### Sequence data

Full-genome sequences of *D. melanogaster* populations were taken from the *Drosophila* Population Genomics Project (DPGP) (http://www.dpgp.org). We used samples from an African and a derived European population whose demography has been reasonably well estimated [43]. The African samples consist of 22 lines from Rwanda including 20 lines from Gikongoro and two from Cyangugu [44]. The European samples consist of four lines from Lyon, France [44] and eight lines from Leiden, the Netherlands [45]. Full-genome sequences were generated by next-generation sequencing of haploid embryos as described in [46]. Consequently, all sequences are haploid, which should minimize the influence of mapping errors. All the lines used for the analysis (22 in Africa and 12 in Europe) were without admixture since in a previous analysis we tested for population substructure using sNMF [47] (A. Wollstein, unpublished results) and excluded these lines from subsequent analysis. This led to the removal of seven out of originally 27 lines from Gikongoro, four out of eight lines from Lyon and two out of 10 lines from Leiden. This procedure coincidentally also removed lines for which genomic blocks of identity-by-descent (IBD) had previously been described [44].

### Statistical analyses

Basic population genetic parameters were estimated using the program *VariScan* [48]. This includes Watterson’s estimator θ_w_ [49] and Tajima’s *D* [50]. Similar to Andrés et al. [4], we used these statistics to search for evidence of balancing selection such as a high level of polymorphisms (θ_w_) and an excess of polymorphisms at intermediate frequency (Tajima’s *D*). Even though these estimators are slightly different from the statistics used in Andrés et al. [4], they look for similar characteristics of balancing selection and are easily computed and simulated. We estimated these statistics for the whole genome and for each chromosome arm (X, 2L, 2R, 3L, 3R) for different window sizes (0.2, 0.5, 1, 2, and 5 kb) to optimize our analysis (see below).

To determine which window size is best, we simulated balancing selection with the software *msms* [51]. We performed coalescent simulations under the estimated demographic model (see below) for a neutral model and a selection model of heterozygote advantage. We set the selection coefficient for heterozygote advantage (*s*) to 0.1. The current effective population size *N*_e_ was estimated from the demographic model as 1.09 × 10^6^ in the European population and 1.62 × 10^6^ in the African population. We used a recombination rate of 0.5 cM/Mb and set the start of selection to 2*N*_e_ generations backward in time. We compared the distributions of the Tajima’s *D* and θ_w_ statistics for simulated data under neutrality and selection and determined the percentage of overlap between the two distributions for each window size (0.2, 0.5, 1, 2, and 5 kb). Windows that show smaller overlap between the distributions should have higher power to distinguish between selection and neutrality.

To conduct a test of balancing selection we estimated the parameters of the demographic null models of the European and African populations (A. Wollstein, unpublished results) based on expectations of the SFS at neutral sites [52]. The SFS for both populations were generated by extracting SNPs located at positions 8 – 30 within small introns (length ≤ 65 bp) as these sites are thought to behave closest to neutrality [53]. Demographic parameters were estimated for a model with instantaneous population size changes at varying time points. The demographic models that best fit the observed data were used for our analysis. The best-fit demographic models allow for a bottleneck in the European population and stepwise growth (with a shallow bottleneck) in the African population. Parameters were estimated for autosomal chromosomes and X chromosome separately as autosomes and sex chromosomes might have different demographic histories [54].

We ran 1000 coalescent simulations for each window of 1-kb across the full genome using *ms* [55] and for each coalescent simulation θ_w_ and Tajima’s *D* were estimated resulting in a neutral distribution of both statistics for each window across the genome. For each window local mutation rates were inferred based on divergence to *D. sechellia* [56, 57]. The local recombination rates were obtained using the *D. melanogaster* recombination rate calculator [58] based on the values of [59]. We then compared the observed values of Tajima’s *D* and θ_w_ for each window to the neutral distributions generated with simulations that take into account the demographic history.

Only those windows for which the observed values of both statistics fell within the upper 95^th^ percentile of the simulations were kept as candidates. A *p*-value was estimated for each window for the θ_w_ and Tajima’s *D* statistics based on the proportion of simulations for which θ_w_ and Tajima’s *D* was greater than the observed value. When the *p*-value was equal to zero, we ran additional 10,000 coalescent simulations to obtain a more precise *p*-value. Benjamini-Hochberg multiple test correction [60] was applied to adjust the *p*-values. Windows with corrected *p*-values < 0.05 were retained as significant.

### GO enrichment analysis

First, a list of genes located in candidate windows, which were significant after correction for multiple testing, was determined for the African and European populations as well as for candidate regions and genes shared between the two populations. Then a gene ontology (GO) enrichment analysis was applied to this list of genes in candidate windows using Cytoscape version 3.2.0 [61], in particular its plugin ClueGO version 2.2.5 (http://apps.cytoscape.org/apps/cluego) and CluePedia version 1.2.5 [62, 63] (http://apps.cytoscape.org/apps/cluepedia). We used Cohen’s Kappa score [64] of 0.7 as a threshold for the proportion of genes shared between enriched ontology and pathway terms to link the terms into GO networks [63] and networks of KEGG [65] and Reactome [66] metabolic pathways. Using ClueGO and CluePedia we integrated enriched GO and pathway terms into networks. Enrichments and depletions of single terms were calculated using a two-tailed hypergeometric test. We applied the false-discovery-rate (FDR) correction [60] and retained the enriched terms with a FDR-corrected *p*-value of less than 0.05 that contained at least three candidate genes, or those whose candidate genes represented at least 4% of the total number of genes related to the term. In addition, we used the option *Fusion* to group the related terms that have similar associated genes.

## Results

### Genome scan

To detect signatures of balancing selection in the genome of *D. melanogaster*, two statistics were used: θ_w_ and Tajima’s *D.* Estimates of these two statistics were computed for windows covering the complete genome. To find an appropriate window size, we performed analyses for various lengths (0.2, 0.5, 1, 2, and 5 kb). Recombination might confine signals of selection to a narrow region. Consequently the analyzed window sizes should be limited. However, windows must also be large enough to contain a sufficient number of polymorphisms for obtaining reasonable estimates of both statistics. We found a window size of 1 kb to be appropriate (see below).

**Fig. 1:**
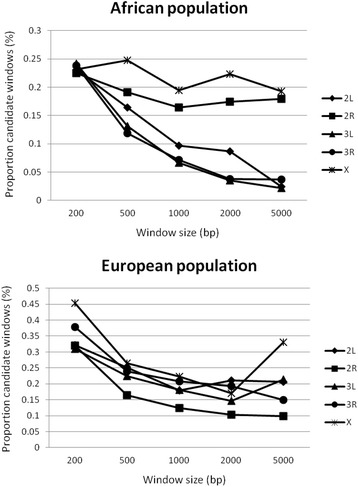
Proportion of candidate windows as a function of window size (in bp) for each chromosome arm (2L, 2R, 3L, 3R and X) in the African and the European populations

We generated empirical distributions of both statistics separately for each of the five major chromosomal arms. Windows in which the two statistics jointly fell into the upper 95^th^ percentile of the distribution were considered as potential candidates of balancing selection. The proportion of windows on each chromosome identified as candidates differed depending on window size, such that the proportions generally decreased with increasing size (Fig. 1). This decrease may be explained by linked recurrent negative [67] or positive selection [68], which is more pronounced in the larger windows. For chromosome arm 2R and X the pattern was not as clear in the African population (Fig. 1). This may be explained by the higher average recombination rate on chromosome arm 2R and X compared to the other chromosome arms [59]. In the European population, we observed an increase for the X chromosome for 5 kb which could be due to the fact that the overall number of windows along the chromosome for 5 kb is low compared to smaller window sizes and consequently the proportion of candidate windows may be inflated purely due to variance. Overall, the proportion of windows we identified as candidates is rather low (<0.25% in Africa). This suggests that our approach may be conservative, which might be influenced by the fact that our two used summary statistics (θ_w_ and Tajima’s *D*) are numerically not independent.

**Fig. 2:**
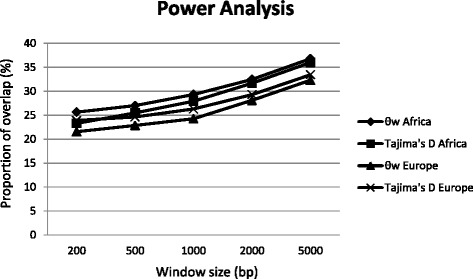
Power analysis for different window sizes (in bp) for the two statistics θ_w_ and Tajima’s *D* for the African and European populations. The overlap between the distributions of simulations with and without selection is represented on the y-axis

In order to examine which window size has the highest power to detect balancing selection, we simulated sequence data under neutrality and balancing selection as described in Methods and compared the overlap between the distributions of neutral and selected Tajima’s *D* and θ_w_ values for different window sizes (Fig. 2). The amount of overlap is inversely related to the power. The more the two distributions overlap, the less ability we have to distinguish selected from putatively neutral regions. We observed for larger window sizes a larger overlap between the two distributions and thus a lower power to distinguish selection from neutrality. The overlap for the 1-kb window is slightly larger than for the 0.2- and 0.5-kb windows and smaller than for the 2- and 5-kb ones. Moreover, the largest difference in power is between 1 kb and 2 kb, which should make 1 kb a good choice. Our choice of window size for subsequent analysis was also influenced by the fact that in Fig. 2 we show perfectly simulated data whereas in our genome scan data may be missing such that the windows behave smaller than the corresponding simulated windows (on average around 10% of the data are missing). Therefore, we continued our analyses with the results obtained from the scan with a window size of 1 kb, even though smaller windows performed slightly better for simulated data.

We observed for the whole genome a mean θ_w_ averaged over all windows of 0.0088 in Africa and 0.0033 in Europe (Table 1). The diversity in the European populations of *D. melanogaster* is reduced on each chromosome arm compared to the African populations, which agrees with previous estimates [44]. Mean Tajima’s *D* is −0.5605 in Africa and −0.4111 in Europe for the whole genome. However, the X chromosome has a reduced Tajima’s *D* in Africa (Tajima’s *D* = −0.8979) compared to the autosomal chromosomes and, on the contrary, an elevated Tajima’s *D* in Europe (Tajima’s *D* = −0.2968). Finally, as previously noticed by Glinka et al. [69], the variance of Tajima’s *D* is much higher in Europe than in Africa (Table 1), which indicates that the European population has been undergoing a bottleneck.

**Table 1:** Statistical values for the mean of θ_W_ and Tajima’s *D* for each chromosome arm and population

Population	Chr.	θ_W_			Tajima's *D*		
		5%	mean	95%	5%	mean	95%
Africa	2 L	0.0028	0.0095	0.0173	−1.4842	−0.4714	0.6342
	2R	0.0022	0.0086	0.0167	−1.5752	−0.5905	0.4894
	3 L	0.0017	0.0088	0.0174	−1.5104	−0.5035	0.6523
	3R	0.0015	0.0069	0.0141	−1.4119	−0.3390	0.8357
	X	0.0033	0.0100	0.0174	−1.7449	−0.8979	0.0263
	Average	0.0102	0.0088	0.0166	−1.2794	−0.5605	0.5980
Europe	2 L	0.0013	0.0034	0.0073	−1.5795	−0.4262	1.1724
	2R	0.0010	0.0036	0.0082	−1.5058	−0.3851	1.1714
	3 L	0.0007	0.0037	0.0087	−1.5849	−0.4517	1.1032
	3R	0.0007	0.0030	0.0071	−1.5830	−0.4957	1.1498
	X	0.0003	0.0030	0.0069	−1.7982	−0.2968	1.4504
	Average	0.0008	0.0033	0.0076	−1.6103	−0.4111	1.1075

### Candidate genes

Since the demographic history of a population can mimic selection (e.g. in the case of a bottleneck), we performed coalescent simulations under the demographic model that best fits the observed data for each population (A. Wollstein, unpublished results). Clearly, the demographic models that we estimated do not represent exactly the history of our populations. Indeed their history is likely more complex but the models fit the data sufficiently well to be used as a null model to reduce the number of false positives. Only windows significantly different from the overall patterns observed in the genome (taking into account the demographic history) are candidates. Then we searched for significantly elevated values of θ_w_ and Tajima’s *D* compared to the distributions obtained by the neutral coalescent simulations that included demography.

We detected 171 candidate windows (of 1 kb each) for the European population and 60 for the African population with significant signatures of balancing selection. Interestingly, in the European population 77 candidate windows are on the X chromosome whereas in the African population we detected only two candidate windows on this chromosome. Then, we identified the genes overlapping with our candidate windows. Occasionally, several genes (up to three for one window) overlapped with the same window. We observed 20 candidate windows in the European population with two genes present (and one with three genes), and eight windows in the African populations. In this case, it was difficult to identify the specific gene under balancing selection. We found 141 (Additional file 1: Table S1) and 45 (Additional file 1: Table S2) candidate genes in the European and African populations, respectively.

We investigated this discrepancy in the number of candidate genes between both populations. In the European population the candidate genes are much larger than in the African population (the average size of the genes is 27.5 kb in Europe and 11.3 kb in Africa). To understand this observation, we studied the genomic distributions of the candidate genes. The European genes are restricted to regions of intermediate to high recombination rates, in which variation is less suppressed by linked selection (discussed above). The 58 candidate genes on the X are distributed over about 20 Mb, whereas those on the autosome arms are located in narrower regions: 16 genes in about 9 Mb on 3R, 16 genes in 13.5 Mb on 3L, 28 genes in 15 Mb on 2R, and 23 genes in 12 Mb on 2L. This pattern may be explained to some extent by the higher average recombination rate on chromosome arm 2R and X compared to the other chromosome arms [59]. The excess of large genes on the X compared to the autosome arms, however, cannot be explained by recombination (“large” is defined somewhat arbitrarily as >10 kb, but other definitions lead to similar conclusions). While 8–10 genes on each autosomal arm are large, 35 are large on the X. This suggests that the excess of large genes on the X in the European population may be due to false positives, which by chance hit longer genes more often than shorter ones. Protein-coding genes generally tend to be longer on the X chromosome compared to autosomes (with average lengths of 8.2 kb vs. 6.1 kb). This may partly explain the observed size distribution between X and autosomes, but needs to be further discussed below.

The average size of the African candidate genes of 11.3 kb is also larger than the average gene length of *D. melanogaster* (which is 6.5 kb for protein-coding genes). This indicates that false positives may play a role in this dataset as well (although to a lesser extent, as only seven out of 45 genes are longer than 10 kb).

**Fig. 3:**
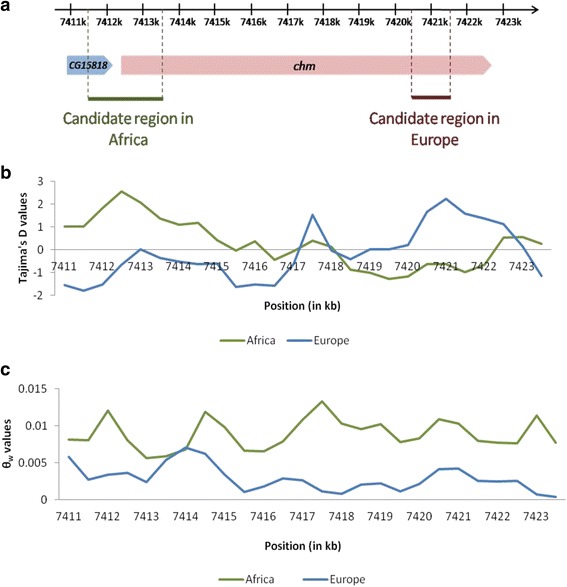
Map of the genes *CG15818* and *chm.* In **a**, the *green* bar represents the region of interest in the African population and the *red* bar represents the region of interest in the European population. In the case of the African population, the region of interest is larger as two contiguous candidate windows are significant. The Tajima’s *D* (**b**) and θ_w_ (**c**) values are plotted for a 1-kb sliding window across the genes *CG15818* and *chm* for both the European and African populations. Plot of θw for a 1-kb window across the gene CG15818 and chm for the European and African populations. The x-axis represents the position on the chromosome and the y-axis, the values of θw. Plot of Tajima\'s D for a 1-kb window across the gene CG15818 and chm for the European and African populations. The x-axis represents the position on the chromosome and the y-axis, the values of Tajima\'s D. Map of the genes CG15818 and chm. the green bar represents the region of interest in the African population and the red bar represents the region of interest in the European population

As mentioned before 43 genes in Europe and 16 genes in Africa are uncertain due to the fact that they are in the same candidate window. Three genes (*fry, chm* and *CG42389*) show signals of balancing selection in both populations (Table 2). However, these signals were detected in two different regions (windows) of the genes (see e.g. Fig. 3 for *chm*). Selection acting in both populations is characteristic for long-term balancing selection, which agrees with our expectation when selection predates the split of the two populations. On the other side, candidate genes with significant statistics only in one population have likely been under more recent balancing selection.

**Table 2:** List of the best candidate genes for the European and African populations with a *p-*value < 10^−4^ for θ_w_ and Tajima’s *D*

Population	FBgn number	Gene name	Chromosome	θ_W_	Tajima's *D*
Europe	FBgn0039004	*Nup133*	3R	0.0029	2.5160
	FBgn0263986	*cd*			
	FBgn0039536	*unc80*	3R	0.0028	2.0467
	FBgn0001316	*klar*	3 L	0.0053	2.1501
	FBgn0265988	*mv*	3 L	0.0046	2.5476
	FBgn0085428	*Nox*	2R	0.0064	2.1896
	FBgn0002543	*lea*	2 L	0.0026	2.4560
	FBgn0031424	*VGlut*	2 L	0.0040	2.2109
	FBgn0085424	*nub*	2 L	0.0064	2.3335
	FBgn0086899	*tlk*	X	0.0044	2.2383
	FBgn0029504	*CHES-1-like*	X	0.0046	2.4145
	FBgn0030244	*CG2157*	X	0.0069	2.3996
	FBgn0030245	*CG1637*			
	FBgn0030286	*CG1657*	X	0.0041	2.0603
	FBgn0267001	*Ten-a*	X	0.0051	2.3024
	FBgn0030412	*Tomosyn*	X	0.0049	2.2223
	FBgn0030466	*CG15744*	X	0.0056	2.2758
			X	0.0058	2.3005
Africa	FBgn0040076	*primo-2*	3R	0.0105	2.2764
	FBgn0040077	*primo-1*			
	FBgn0039519	*Cyp6a18*	3R	0.0130	2.1106
	FBgn0036173	*CG7394*	3 L	0.0103	2.1201
	FBgn0261853	*CG42782*	2R	0.0205	1.9016
	FBgn0031910	*CG15818*	2 L	0.0121	1.8187
	FBgn0028387	*chm*	2 L	0.0080	2.5511
	FBgn0028899	*CG31817*	2 L	0.0144	1.8100
	FBgn0259735	*CG42389*	2 L	0.0202	1.1303

The number of candidate genes detected in the two populations is very different: 45 in the African population and 141 in the European population. The differences between both populations are even more striking on the X chromosome where we found 58 candidate genes (overlapping with 77 windows) in Europe and only one candidate gene (overlapping with two windows) in Africa. In converse, this would mean that on the autosomes the total numbers are relatively similar: 44 in Africa and 83 in Europe. The disparity in the number of candidate genes between populations is unlikely strongly influenced by differences in statistical power as the proportion of overlap between simulated selected and neutral data in Africa for a 1-kb window is not much different from Europe (Fig. 2). However, in Africa the proportion of overlap is slightly higher, which might indicate a lower power than in Europe. Many genes significant in Europe show high values of Tajima’s *D* and θ_w_ in Africa as well but they do not reach statistical significance in this population. In Europe, all the significant windows where we found our candidate genes have also significant θ_w_ values in Africa, but their Tajima’s *D* values are not significant. In the African population, we observed 13 genes (same windows in Africa and Europe) with a Tajima’s *D* > 0 (*p-*values are going from 0.24 to 0.82) for the X chromosome and 18 candidate genes with a Tajima’s *D* > 0.5 (*p-*values = 1) for the autosomal chromosomes.

To summarize, taking into account possible false positives the number of candidate genes on the X (without the excess of large genes) converges toward the numbers of candidate genes on the autosome arms in the European population. This is particularly the case for chromosome arm 2R. Furthermore, the overall number of candidate genes in the European population is no longer much greater than that of the African population and the numbers on the autosomes of both populations are more similar than reported above. On the other hand, the African X and the European X still differ greatly in the number of candidate genes, which might be due to an increase of false positives on the European X (discussed below).

### GO terms

A GO analysis was performed on significant genes to determine the groups of terms enriched for both the European and the African populations. Groups are based on GO hierarchy or on the kappa score [64], which is based on the overlapping genes (within categories). The name of the group is determined by the most significant term of the group. The European population is enriched for many terms, 41 biological function categories are enriched and are grouped in eight terms (see Additional file 1: Table S3). We observed three groups with many GO terms and consequently have a high number of genes including shared genes between several GO terms (Additional file 1: Table S3). Additionally, we observed three GO terms enriched within the molecular process and cellular component categories each (Additional file 1: Table S4). Finally, eight pathways from the KEGG [65] and Reactome [66] databases were enriched for candidate genes (Additional file 1: Table S4). However, since half of the European candidate genes are located on the X chromosome and there is evidence that these genes may contain an increased number of false positives we repeated our GO analysis with autosomal genes only. With this reduced dataset we only found four enriched GO terms under biological process (*mushroom body development*, *regulation of circadian sleep/wake cycle*, *organophosphate metabolic process* and *nucleotide metabolic process*) and *transcription cofactor activity* under molecular process. All five of these terms were also significant in the original analysis. Analyzing the complete African candidate gene set we found only two GO terms enriched (*aspartic-type endopeptidase activity* and *surfactant metabolism*) for all categories. However, this result might be an artifact since the three genes enriched for these terms (*CG31928, CG31926* and *CG33128*) are physically adjacent, which might explain why they collectively show a signal of balancing selection.

Then we examined in greater detail the more extreme candidate genes (*p-*value < 10^−4^ for θ_w_ and Tajima’s *D* after multiple testing corrections). In the European population, we found 17 genes (Table 2), which include genes involved in different functions such as circadian rhythm (*unc80*), cell migration (*klar*), neuronal development (*lea* and *Ten-a*), neurogenesis and memory (*Tomosyn*), and chemical synaptic transmission (*VGlut*). We also found genes related to immunity (*Nox, nub* and *tlk*) or involved in phagocytosis (*mv* and *CHES-1-like)*. The genes *cd* and *Nup133* are located in the same genomic region; *cd* is involved in several processes including response to oxidative stress, while *Nup133* is involved in nucleocytoplasmic transporter activity. Interestingly, there is evidence that *Nup133* may also have undergone recurrent adaptive evolution in *D. simulans and D. melanogaster* [70]. Candidate genes with unknown functions have been also found (*CG2157, CG1637, CG1657* and *CG15744*). Concerning the African population, 9 genes (Table 2) are highly significant (*p-*values < 10^−4^). However, the genes *primo-1* and *primo-2* are located in the same region. Their proteins have the same function. The genes *CG15818* and c*hm* are also localized in the same region with two significant windows adjacent. The gene *chm* is involved in 15 biological processes such as neuron differentiation, development (larvae, pupal and wing), histone acetylation and regulation of metabolic processes. The gene *Cyp6a18* has an oxidoreductase activity. However, the function of the other genes remains unknown. Moreover, two of the best candidate genes (*chm* and *CG42389*) in Rwanda are also significant in Europe (Table 3). In addition, the gene *fry* is also shared by the two populations.

**Table 3:** List of candidate genes shared by the African and European populations and the values of the significant statistics observed (*p-*value < 0.05) for θ_w_ and Tajima’s *D*

FBgn number	Gene name	Chromosome	Population	θ_W_	Tajima's D
FBgn0016081	*fry*	3 L	Europe	0.0051 (0.0121)	2.1338 (<10^−4^)
			Africa	0.0103 (<10^−4^)	1.8448 (0.0219)
FBgn0028387	*chm*	2 L	Europe	0.0042 (0.0285)	2.2383 (0.0231)
			Africa	0.008 (<10^−4^)	2.5511 (<10^−4^)
FBgn0259735	*CG42389*	2 L	Europe	0.0025 (0.0469)	2.3025 (<10^−4^)
			Africa	0.0202 (<10^−4^)	1.1303 (<10^−4^)

The *p-*values of the statistics are indicated in the parentheses

## Discussion

Some recent studies have found new examples of genes under balancing selection in *D. melanogaster.* For instance, Sato et al. [37] detected balancing selection in core promoter regions. Unckless et al. [36] found evidence of alleles maintained by balancing selection in genes encoding antimicrobial peptides. Nonetheless, examples of genes under balancing selection in *D. melanogaster* are still scarce, and no genome-wide analysis for balancing selection has been done in this species with the exception of our own previous preliminary work [8]. Based on the availability of a wealth of NGS data we approached the difficulties of detecting balancing selection in the genome using two common statistics (high genetic diversity and intermediate-frequency polymorphism), without specifying a certain type of balancing selection. These two features are characteristics of balancing selection and cannot be confounded by other types of selection such as purifying and positive directional selection. Furthermore, we accounted for demography in our methods. Thus we followed a similar approach as Andrés et al. [4] rather than using a model-based method, such as DeGiorgio et al. [71].

In total, we found 183 candidate genes: 141 in the European population and 45 in the African one, with only three genes overlapping between both populations*.* This overlap is much smaller than found in humans between a Europe-derived and an Africa-derived population [4], which may be explained by the much longer separation time (in generations) between the two fly populations. However, even if the *p*-values are not significant, we observed many genes with high estimates of θ_w_ and Tajima’s *D* shared between both populations. The small overlap of significant genes might therefore be due to our statistical approach being conservative.

Although we fitted demographic models to the data for both the African and European populations, we found evidence for false positives in our set of candidate genes. More false positives appear to be present in the European set of candidate genes (in particular on the X chromosome). Inaccuracies in the estimation of demographic parameters may be the primary reason for this problem. We estimated demography for the X and the autosomes separately, based on the SFS at neutral sites [52, 53]. Since the European X chromosome harbors the lowest amount of variability the estimated demography might have been less precise for the European X compared to the European autosomes and the African X and autosomes, leading to an elevation of false positives.

The discrepancy between the X chromosomes of both populations is particularly large. We observed only one candidate gene on the African X, but 20–30 on the European one (after correcting for the excess of large genes). As mentioned above, all significant windows on the X where we found our candidate genes in Europe have also significant θ_w_ values in Africa, but their Tajima’s *D* values are not significant. The reason for this may be as follows. As already Glinka et al. [69] noticed, the variance of the European X is higher than that under standard neutrality, and lower in Africa (see also Table 1). Therefore, scaling Tajima’s *D* with the standard neutral variance may have led to too many candidates in Europe and/or too few in Africa.

Concerning the functions of genes, we observed enrichment in many biological processes in the European population. When we repeated the analysis for autosomal genes only, we were, however, only left with five GO terms. This reduction might be because of an overrepresentation of certain functions on the X chromosome, but could also be purely due to reduced statistical power given the lower number of genes in the autosomal dataset. GO terms that were consistently detected include ones related to circadian behavior and the development of mushroom bodies. Mushroom bodies play a major role in olfactory learning and memory, but have also been shown to be involved in other behavioral traits and the regulation of sleep [72, 73]. Even though these GO terms seem to be closely related their statistical significance is driven by different sets of genes (Additional file 1: Table S3). Candidate genes related to neuronal development and behavior are particularly interesting, as evidence of balancing selection in genes associated with neuromuscular junction development and behavior [35] has previously been reported. For the African population, only two GO terms are enriched and the three corresponding genes lie in the same genomic region.

Andrés et al. [4] performed a genome-wide analysis to detect balancing selection in humans using a similar method. They found a relatively high number of candidate genes related to immunity. Indeed, genes involved in immune defense are assumed to often evolve under balancing selection. However, we did not find an enrichment of genes involved in immunity. Only a few candidate genes of our scan are involved in immunity, such as *Ser* and *tlk* genes in Europe and *Dif* gene in Africa. However, we detected four genes involved in wound healing (*Cad96Ca, Fhos, Rok* and *Hml*) in Europe. Even if the majority of our candidate genes seem to be involved in other functions, they could also play a role during an infection. It has been shown that the immune system is linked to circadian rhythms [74]. Clock genes may be involved in the fight against bacterial invasion [75, 76]. For example, the ortholog of our candidate gene *cry* has been shown to up-regulate pro-inflammatory cytokine gene expression during an infection in mice [77]. Moreover, an enrichment of genes involved in extracellular matrix interaction (Additional file 1: Table S4) has been found. Andrés et al. [4] also detected candidate genes involved in the extracellular matrix. Concerning the examples found previously in *D. melanogaster* [35–37]*,* we did not confirm any of these examples although we observed some genes with similar functions such as oxidation-reduction process and olfactory behavior. Furthermore, Comeron et al. [35] found a P450 gene (*Cyp6a16*) as candidate gene for balancing selection, which was also detected in our search. The fact that we did not find overlap with other studies might be explained by the difference in the methods [35] and the samples, which are not exactly the same. Moreover, in the studies [36] and [37], the authors look for balancing selection only in a small part of the genome of *D. melanogaster*. We also find little overlap when we compare the results to our earlier study [8]: only one gene (*CG18208*) is shared for the African candidate genes. This discrepancy might again be explained by our different methodology and different dataset. In our previous study, we preselected windows in which the observed θ_w_ and Tajima’s *D* values jointly fell within the 95^th^ percentile of the empirical distribution for each chromosome. Consequently, many candidate windows were removed. Moreover, in our current study we performed rigorous multiple testing correction, which led to many overlapping genes losing significance after correction.

We found 17 extreme genes (*p-*value < 10^−4^ for θ_w_ and Tajima’s *D*) in the European population and 9 in the African population. Among these genes, some are related to immunity. The gene *tlk* has been reported to be involved in the humoral immune response [78]. The gene *Nox* has a role in both regulation of the gut microbiota and resistance to infection by inducing the generation of reactive oxygen species [79]. The gene *nub* is a negative regulator of antimicrobial peptide biosynthesis. It represses the expression of NF-κB-dependent immune genes and increases the tolerance to gut microbiota [80]. The gene *CHES-1-like* is required for phagocytosis of the fungal pathogen *Candida olbicans* [81]*. CG15818* has been shown to be down-regulated in flies infected by the Nora virus [82]. The gene *Cyp6a18* may play a role in the metabolism of insect hormones and in the resistance to insecticides. Concerning the other genes, some are involved in neural function. The gene *Ten-a* is involved in neuronal development and also in the establishment of neuron connectivity [83]. The gene *Tomosyn* plays a role in the regulation of behavioral plasticity and memory [84] and *VGlut* is involved in neuromuscular junctions. The genes *primo-1* and *primo-2* have both a function in dephosphorylation and play a role in different functions such as neurogenesis [85]. Finally, *chm* enhances JNK signaling during metamorphosis and thorax closure and acts positively in the JNK-dependent apoptotic pathway [86]. This gene is also required for the maintenance of Hox gene silencing by PolyComb group proteins [87]. It is interesting that many genes have a role in the nervous system, which is known to be connected to immunity in insects [88]. While many examples of balancing selection have been found in immune genes, genes involved in other functions might be under this type of selection due to temporal changes in the environment like fluctuations between seasons [89].

Thus, of the extreme genes, many seem to be related to immunity or to neuronal function. Of course, it will be interesting to examine these genes in more detail. In addition to our analysis, it would be informative to combine our summary statistics with other estimators, such as linkage disequilibrium to detect evidence for recent balancing selection, and species comparisons to find trans-species polymorphism as signatures of ancient balancing selection.

## Conclusion

We identified candidate genes under balancing selection in two populations of *D. melanogaster*: 141 in the European population and 45 in the African one*.* The difference between both populations is mainly due to an excess of candidate genes on the European X chromosome, which is likely due to false positives. Correcting for this effect reduces the difference between both populations considerably. Among the candidate genes detected in the European population there is an overrepresentation of genes involved in neuronal development and circadian rhythm. Other genes are involved in immunity including the top candidates. These top genes are also involved in behavioral plasticity, memory, neuromuscular junctions or neurogenesis.

## Additional file

Additional file 1: Table S1. List of candidate genes for the European population and the values of the significant statistics observed (*p-*value < 0.05) for a 1-kb window. The *p-*values of the statistics are indicated in the parentheses. **Table S2**: List of candidate genes for the African population and the values of the significant statistics observed (*p-*value < 0.05) for a 1-kb window. The *p-*value of the statistics is indicated in the brackets. **Table S3**: List of enriched GO terms and the genes grouped under this term for the European population in biological processes. The GO term shown in bold is the name of the group (the most significant term of the group). **Table S4**: List of enriched GO terms and the genes grouped under this term for the European population for molecular function, cellular component and KEGG and Reactome pathways. (DOCX 59 kb)

## Abbreviations

FDR: False-discovery rate
GO: Gene ontology
MHC: Major histocompatibility complex
NGS: Next-generation sequencing
SFS: Site frequency spectrum
SNP: Single nucleotide polymorphism
