# Supplementary material for: A genome-wide scan for genes under balancing selection in Drosophila melanogaster
Source: BMC Evol Biol. 2017 Jan 13;17:15. doi: 10.1186/s12862-016-0857-z (PMC5237213; doi:10.1186/s12862-016-0857-z)
Supplement: Additional file 1: Table S1. — List of candidate genes for the European population and the values of the significant statistics observed (p-value < 0.05) for a 1-kb window. The p-values of the statistics are indicated in the parentheses. Table S2: List of candidate genes for the African population and the values of the significant statistics observed (p-value < 0.05) for a 1-kb window. The p-value of the statistics is indicated in the brackets. Table S3: List of enriched GO terms and the genes grouped under this term for the European population in biological processes. The GO term shown in bold is the name of the group (the most significant term of the group). Table S4: List of enriched GO terms and the genes grouped under this term for the European population for molecular function, cellular component and KEGG and Reactome pathways. (DOCX 59 kb) [file 12862_2016_857_MOESM1_ESM.docx]

**Additional file**

**Table S1:** List of candidate genes for the European population and the values of the significant statistics observed (*p-*value < 0.05) for a 1-kb window. The *p-*values of the statistics are indicated in the parentheses.

| **FBgn number** | **Gene name** | **Chromosome** | **θ_W_** | | **Tajima's D** | |
| --- | --- | --- | --- | --- | --- | --- |
| FBgn0039004 | *Nup133* | 3R | 0.0029 | (<10^-4^) | 2.5160 | (<10^-4^) |
| FBgn0263986 | *cd* |  |  |  |  |  |
| FBgn0039536 | *unc80* | 3R | 0.0028 | (<10^-4^) | 2.0467 | (<10^-4^) |
| FBgn0001316 | *klar* | 3L | 0.0053 | (<10^-4^) | 2.1501 | (<10^-4^) |
| FBgn0265988 | *mv* | 3L | 0.0046 | (<10^-4^) | 2.5476 | (<10^-4^) |
| FBgn0085428 | *Nox* | 2R | 0.0064 | (<10^-4^) | 2.1896 | (<10^-4^) |
| FBgn0002543 | *lea* | 2L | 0.0026 | (<10^-4^) | 2.4560 | (<10^-4^) |
| FBgn0031424 | *VGlut* | 2L | 0.0038 | (0.0023) | 2.0871 | (0.0444) |
|  |  |  | 0.0040 | (<10^-4^) | 2.2109 | (<10^-4^) |
| FBgn0085424 | *nub* | 2L | 0.0064 | (<10^-4^) | 2.3335 | (<10^-4^) |
| FBgn0086899 | *tlk* | X | 0.0044 | (<10^-4^) | 2.2383 | (<10^-4^) |
| FBgn0029504 | *CHES-1-like* | X | 0.0046 | (<10^-4^) | 2.4145 | (<10^-4^) |
|  |  |  | 0.0028 | (0.0374) | 2.2258 | (0.0107) |
| FBgn0030244 | *CG2157* | X | 0.0069 | (<10^-4^) | 2.3996 | (<10^-4^) |
| FBgn0030245 | *CG1637* |  |  | (<10^-4^) |  | (<10^-4^) |
| FBgn0030286 | *CG1657* | X | 0.0042 | (<10^-4^) | 1.9313 | (0.0413) |
|  |  |  | 0.0041 | (<10^-4^) | 2.0603 | (<10^-4^) |
| FBgn0267001 | *Ten-a* | X | 0.0051 | (<10^-4^) | 2.3024 | (<10^-4^) |
| FBgn0030412 | *Tomosyn* | X | 0.0049 | (<10^-4^) | 2.2223 | (<10^-4^) |
| FBgn0030466 | *CG15744* | X | 0.0056 | (<10^-4^) | 2.2758 | (<10^-4^) |
|  |  |  | 0.0046 | (0.0005) | 2.1004 | (0.0107) |
|  |  |  | 0.0058 | (<10^-4^) | 2.3005 | (<10^-4^) |
| FBgn0086680 | *vvl* | 3L | 0.0016 | (0.0004) | 1.8337 | (<10^-4^) |
| FBgn0013765 | *cnn* | 2R | 0.0052 | (0.0004) | 2.0192 | (<10^-4^) |
| FBgn0050062 | *CG30062* |  |  |  |  |  |
| FBgn0024315 | *Picot* | 2R | 0.0075 | (0.0004) | 2.1610 | (<10^-4^) |
| FBgn0024319 | *Nach* |  |  |  |  |  |
| FBgn0024992 | *CG2658* | X | 0.0055 | (0.0005) | 2.2549 | (<10^-4^) |
| FBgn0052791 | *CG32791* | X | 0.0069 | (0.0005) | 2.2098 | (<10^-4^) |
| FBgn0052792 | *ppk8* |  |  |  |  |  |
| FBgn0030613 | *Rab3-GEF* | X | 0.0029 | (0.0399) | 2.4475 | (<10^-4^) |
|  |  |  | 0.0037 | (0.0005) | 2.3121 | (<10^-4^) |
|  |  |  | 0.0039 | (0.0011) | 2.3986 | (<10^-4^) |
| FBgn0260486 | *Ziz* | 2L | 0.0041 | (0.0006) | 2.2383 | (<10^-4^) |
|  |  |  | 0.0034 | (0.0375) | 2.3238 | (<10^-4^) |
| FBgn0261041 | *stj* | 2R | 0.0095 | (0.0009) | 1.8089 | (<10^-4^) |
| FBgn0030081 | *CG7246* | X | 0.0042 | (0.0011) | 2.3344 | (<10^-4^) |
| FBgn0034599 | *hng1* | 2R | 0.0068 | (0.0013) | 2.4741 | (<10^-4^) |
| FBgn0029896 | *CG3168* | X | 0.0048 | (0.0017) | 2.3344 | (<10^-4^) |
| FBgn0261260 | *mgl* | X | 0.0036 | (0.0017) | 2.4179 | (<10^-4^) |
|  |  |  | 0.0060 | (0.0047) | 2.0536 | (0.0107) |
| FBgn0000028 | *acj6* | X | 0.0074 | (0.0017) | 2.2739 | (<10^-4^) |
| FBgn0023506 | *Es2* | X | 0.0031 | (0.0415) | 2.2556 | (<10^-4^) |
| FBgn0023506 | *Es2* | X | 0.0042 | (0.0028) | 2.4465 | (<10^-4^) |
| FBgn0014032 | *Sptr* |  |  |  |  |  |
| FBgn0025378 | *CG3795* | X | 0.0023 | (0.0040) | 2.3519 | (<10^-4^) |
| FBgn0001624 | *dlg1* | X | 0.0036 | (0.00457) | 2.6062 | (<10^-4^) |
|  |  |  | 0.0033 | (0.0090) | 2.4413 | (<10^-4^) |
| FBgn0051145 | *CG31145* | 3R | 0.0050 | (0.0045) | 2.3290 | (<10^-4^) |
| FBgn0262733 | *Src64B* | 3L | 0.0039 | (0.0053) | 2.4556 | (<10^-4^) |
| FBgn0025833 | *CG8910* | 2R | 0.0082 | (0.00553) | 1.7806 | (<10^-4^) |
| FBgn0050263 | *CG30263* | 2R | 0.0039 | (0.0059) | 2.3238 | (<10^-4^) |
| FBgn0030884 | *CG6847* | X | 0.0035 | (0.0066) | 2.3050 | (<10^-4^) |
| FBgn0263111 | *cac* | X | 0.0031 | (0.00902) | 2.1962 | (<10^-4^) |
|  |  |  | 0.0032 | (0.0085) | 2.3175 | (<10^-4^) |
| FBgn0262872 | *milt* | 2L | 0.0027 | (0.00881) | 2.3792 | (<10^-4^) |
| FBgn0020653 | *Trxr-1* | X | 0.0035 | (0.0090) | 2.4556 | (<10^-4^) |
| FBgn0004168 | *5-HT1A* | 2R | 0.0033 | (0.0104) | 2.5858 | (<10^-4^) |
| FBgn0050295 | *Ipk1* | 2R | 0.0045 | (0.0104) | 2.1563 | (<10^-4^) |
| FBgn0038880 | *SIFaR* | 3R | 0.0049 | (0.0112) | 2.6636 | (<10^-4^) |
| FBgn0016081 | *fry* | 3L | 0.0051 | (0.0120) | 2.1338 | (<10^-4^) |
| FBgn0051774 | *fred* | 2L | 0.0043 | (0.01230) | 2.4333 | (<10^-4^) |
| FBgn0028369 | *CG3603* | X | 0.0074 | (0.00057) | 2.1427 | (0.0413) |
| FBgn0028369 | *kirre* |  |  |  |  |  |
| FBgn0028369 | *kirre* | X | 0.0042 | (0.01250) | 2.1582 | (<10^-4^) |
| FBgn0003463 | *sog* | X | 0.0046 | (<10^-4^) | 2.0855 | (0.0413) |
|  |  |  | 0.0031 | (0.0137) | 2.3381 | (<10^-4^) |
| FBgn0027093 | *Aats-arg* | X | 0.0031 | (0.0141) | 2.5032 | (<10^-4^) |
|  |  |  | 0.0042 | (0.0343) | 2.6227 | (<10^-4^) |
| FBgn0262719 | *CG43163* | 3L | 0.0043 | (0.0148) | 2.1742 | (<10^-4^) |
| FBgn0017561 | *Ork1* | X | 0.0031 | (0.0149) | 2.4048 | (<10^-4^) |
| FBgn0267253 | *CG32700* | X | 0.0046 | (0.0152) | 2.3665 | (<10^-4^) |
| FBgn0004197 | *Ser* | 3R | 0.0059 | (0.0156) | 2.2602 | (<10^-4^) |
| FBgn0034974 | *CG16786* | 2R | 0.0055 | (0.0164) | 2.2882 | (<10^-4^) |
| FBgn0086129 | *snama* |  |  |  |  |  |
| FBgn0029167 | *Hml* | 3L | 0.0051 | (0.00467) | 2.3466 | (0.0414) |
|  |  |  | 0.0043 | (0.0173) | 2.6227 | (<10^-4^) |
| FBgn0053223 | *CG33223* | X | 0.0070 | (0.01836) | 2.5690 | (<10^-4^) |
| FBgn0030041 | *CG12116* |  |  |  |  |  |
| FBgn0025741 | *PlexA* | X | 0.0079 | (0.0187) | 2.4014 | (<10^-4^) |
|  |  |  | 0.0030 | (0.0454) | 2.4018 | (<10^-4^) |
| FBgn0027505 | *Rab3-GAP* | 2L | 0.0034 | (0.0197) | 2.2297 | (<10^-4^) |
| FBgn0263512 | *Vsx2* | X | 0.0032 | (0.0198) | 2.4703 | (<10^-4^) |
| FBgn0030055 | *CG12772* | X | 0.0033 | (0.0226) | 2.4413 | (<10^-4^) |
| FBgn0086757 | *cbs* | 2R | 0.0026 | (0.0240) | 2.1234 | (<10^-4^) |
| FBgn0024973 | *CG2701* | X | 0.0044 | (0.0240) | 2.4367 | (<10^-4^) |
| FBgn0023458 | *Rbcn-3A* | X | 0.0020 | (0.0241) | 2.2509 | (<10^-4^) |
| FBgn0031263 | *CG2789* | 2L | 0.0062 | (0.0243) | 2.4113 | (<10^-4^) |
| FBgn0085446 | *CG34417* | X | 0.0034 | (0.0248) | 2.4620 | (<10^-4^) |
| FBgn0039225 | *Ets96B* | 3R | 0.0052 | (0.0249) | 2.6073 | (<10^-4^) |
| FBgn0030011 | *Gbeta5* | X | 0.0038 | (0.0251) | 2.3803 | (<10^-4^) |
| FBgn0261509 | *haf* | 2L | 0.0039 | (0.0271) | 2.4703 | (<10^-4^) |
| FBgn0051935 | *CG31935* |  |  |  |  |  |
| FBgn0027496 | *epsilonCOP* | 2L | 0.0043 | (0.0271) | 2.1422 | (<10^-4^) |
| FBgn0038727 | *CG7432* | 3R | 0.0025 | (0.0272) | 2.0723 | (<10^-4^) |
| FBgn0261931 | *CG42797* | X | 0.0049 | (0.0279) | 2.4626 | (<10^-4^) |
| FBgn0033766 | *CG8771* | 2R | 0.0034 | (0.0283) | 2.2862 | (<10^-4^) |
| FBgn0003380 | *Sh* | X | 0.0045 | (0.0289) | 2.5907 | (<10^-4^) |
| FBgn0000259 | *CkIIbeta* | X | 0.0042 | (0.0319) | 2.3050 | (<10^-4^) |
| FBgn0029922 | *CG14431* | X | 0.0044 | (0.0323) | 2.4465 | (<10^-4^) |
| FBgn0052732 | *CG32732* |  |  |  |  |  |
| FBgn0261985 | *Ptpmeg* | 3L | 0.0044 | (0.0336) | 1.9364 | (<10^-4^) |
| FBgn0035131 | *mthl9* |  |  |  |  |  |
| FBgn0050395 | *CG30395* | 2R | 0.0052 | (0.0363) | 1.9662 | (<10^-4^) |
| FBgn0023524 | *CG3078* | X | 0.0024 | (0.0369) | 2.3025 | (<10^-4^) |
| FBgn0000064 | *Ald* | 3R | 0.0065 | (0.0374) | 2.1660 | (<10^-4^) |
| FBgn0003301 | *rut* | X | 0.0030 | (0.0390) | 2.3333 | (<10^-4^) |
| FBgn0011589 | *Elk* | 2R | 0.0039 | (0.0432) | 2.0525 | (<10^-4^) |
| FBgn0265597 | *rad* | X | 0.0028 | (0.0448) | 2.5388 | (<10^-4^) |
| FBgn0259735 | *CG42389* | 2L | 0.0025 | (0.0469) | 2.3025 | (<10^-4^) |
| FBgn0011653 | *mas* | 3L | 0.0022 | (0.0485) | 2.1484 | (<10^-4^) |
| FBgn0004657 | *mys* | X | 0.0036 | (0.0486) | 2.2485 | (<10^-4^) |
| FBgn0260439 | *Pp2A-29B* | 2L | 0.0031 | (0.0494) | 2.2969 | (<10^-4^) |
| FBgn0034598 | *CG4266* | 2R | 0.0051 | (0.0499) | 2.3859 | (<10^-4^) |
| FBgn0030274 | *Lint-1* | X | 0.0052 | (0.0017) | 2.2453 | (0.0107) |
| FBgn0000479 | *dnc* | X | 0.0044 | (0.0489) | 2.0942 | (0.0107) |
| FBgn0029881 | *pigs* | X | 0.0054 | (<10^-4^) | 2.0352 | (0.0211) |
| FBgn0026181 | *Rok* | X | 0.0040 | (<10^-4^) | 2.1217 | (0.0211) |
| FBgn0004045 | *Yp1* | X | 0.0056 | (0.0005) | 2.1276 | (0.0211) |
| FBgn0030174 | *CG15312* |  |  |  |  |  |
| FBgn0266350 | *CG12535* | X | 0.0042 | (0.0223) | 2.1920 | (0.0211) |
| FBgn0264979 | *CG4267* | 2L | 0.0044 | (0.0058) | 2.0781 | (0.0230) |
| FBgn0260933 | *rempA* | 2L | 0.0085 | (0.0155) | 2.2570 | (0.0230) |
| FBgn0032085 | *CG9555* | 2L | 0.0078 | (0.0186) | 1.9049 | (0.0230) |
| FBgn0013746 | *alien* |  |  |  |  |  |
| FBgn0032086 | *CG17906* |  |  |  |  |  |
| FBgn0028387 | *chm* | 2L | 0.0042 | (0.0284) | 2.2383 | (0.0230) |
| FBgn0034229 | *CG4847* | 2R | 0.0054 | (0.0039) | 1.9804 | (0.0241) |
| FBgn0034230 | *CG4853* |  |  |  |  |  |
| FBgn0034776 | *CG13527* | 2R | 0.0081 | (0.0039) | 1.5816 | (0.0241) |
| FBgn0259145 | *CG42260* |  |  |  |  |  |
| FBgn0028473 | *CG8801* | 2R | 0.0035 | (0.0188) | 2.1318 | (0.0241) |
| FBgn0033408 | *CG8800* |  |  |  |  |  |
| FBgn0034282 | *Mapmodulin* | 2R | 0.0066 | (0.0246) | 2.1790 | (0.0241) |
| FBgn0039234 | *nct* | 3R | 0.0072 | (<10^-4^) | 2.1898 | (0.0308) |
| FBgn0025680 | *cry* | 3R | 0.0047 | (0.0112) | 2.2047 | (0.0308) |
|  |  |  | 0.0075 | (0.0015) | 2.1369 | (0.0308) |
| FBgn0038660 | *CG14291* | 3R | 0.0083 | (0.0022) | 2.0666 | (0.0308) |
| FBgn0261262 | *CG42613* | 3R | 0.0024 | (0.0197) | 2.0147 | (0.0308) |
| FBgn0263983 | *CG43732* |  |  |  |  |  |
| FBgn0022800 | *Cad96Ca* | 3R | 0.0051 | (0.0374) | 2.3091 | (0.0308) |
| FBgn0039290 | *CG13654* |  |  |  |  |  |
| FBgn0029688 | *lva* | X | 0.0045 | (<10^-4^) | 2.0109 | (0.0313) |
| FBgn0266199 | *CG43902* | X | 0.0045 | (<10^-4^) | 2.0621 | (0.0313) |
| FBgn0000709 | *fliI* | X | 0.0039 | (0.0265) | 2.1920 | (0.03138) |
| FBgn0029939 | *CG9650* | X | 0.0036 | (0.0116) | 2.1544 | (0.0413) |
| FBgn0036022 | *CG8329* | 3L | 0.0062 | (<10^-4^) | 2.1894 | (0.0414) |
| FBgn0262524 | *ver* | 3L | 0.0049 | (0.0008) | 2.1153 | (0.0414) |
| FBgn0004926 | *eIF-2beta* |  |  |  |  |  |
| FBgn0010825 | *Gug* | 3L | 0.0042 | (0.0035) | 2.1390 | (0.04144) |
| FBgn0052062 | *A2bp1* | 3L | 0.0080 | (0.0095) | 2.2025 | (0.0414) |
| FBgn0266084 | *Fhos* | 3L | 0.0032 | (0.0245) | 2.2143 | (0.0414) |
| FBgn0264815 | *Pde1c* | 2L | 0.0061 | (<10^-4^) | 2.2595 | (0.0444) |
| FBgn0032382 | *Mal-B2* | 2L | 0.0050 | (<10^-4^) | 2.1477 | (0.0444) |
| FBgn0032036 | *CG13384* | 2L | 0.0042 | (0.0023) | 2.0621 | (0.0444) |
| FBgn0262001 | *CG42819* |  |  |  |  |  |
| FBgn0016059 | *Sema-1b* | 2R | 0.0055 | (<10^-4^) | 1.8828 | (0.0471) |
| FBgn0003545 | *sub* | 2R | 0.0085 | (<10^-4^) | 1.9183 | (0.0471) |
| FBgn0010434 | *cora* | 2R | 0.0049 | (<10^-4^) | 2.0301 | (0.0471) |

**Table S2:** List of candidate genes for the African population and the values of the significant statistics observed (*p-*value < 0.05) for a 1-kb window. The *p-*value of the statistics is indicated in the brackets.

| **FBgn number** | **Gene name** | **Chromosome** | **θ_W_** | | **Tajima's D** | |
| --- | --- | --- | --- | --- | --- | --- |
| FBgn0040076 | *primo-2* | 3R | 0.0105033 | (<10^-4^) | 2.2763961 | (<10^-4^) |
| FBgn0040077 | *primo-1* |  |  |  |  |  |
| FBgn0039519 | *Cyp6a18* | 3R | 0.0130491 | (<10^-4^) | 2.1106114 | (<10^-4^) |
| FBgn0036173 | *CG7394* | 3L | 0.0102946 | (<10^-4^) | 2.1201439 | (<10^-4^) |
| FBgn0261853 | *CG42782* | 2R | 0.0204579 | (<10^-4^) | 1.9015773 | (<10^-4^) |
| FBgn0031910 | *CG15818* | 2L | 0.0120518 | (<10^-4^) | 1.8186764 | (<10^-4^) |
| FBgn0028387 | *chm* | 2L | 0.0080177 | (<10^-4^) | 2.5510996 | (<10^-4^) |
| FBgn0028899 | *CG31817* | 2L | 0.0143639 | (<10^-4^) | 1.8100277 | (<10^-4^) |
| FBgn0259735 | *CG42389* | 2L | 0.0202475 | (<10^-4^) | 1.1303249 | (<10^-4^) |
| FBgn0036817 | *CG6865* | 3L | 0.0132736 | (0.0001) | 1.8804494 | (<10^-4^) |
| FBgn0001258 | *ImpL3* | 3L | 0.0155864 | (0.0003) | 1.6712532 | (<10^-4^) |
| FBgn0051469 | *CG31469* | 3R | 0.007719 | (0.0004) | 2.3374541 | (<10^-4^) |
| FBgn0033732 | *CG13157* | 2R | 0.0184698 | (0.0030) | 1.3831235 | (<10^-4^) |
| FBgn0033480 | *mRpL42* | 2R | 0.0049978 | (0.0038) | 2.4522424 | (<10^-4^) |
| FBgn0013435 | *cdc2rk* |  |  |  |  |  |
| FBgn0038938 | *CG7084* | 3R | 0.0084234 | (0.0043) | 1.9567057 | (<10^-4^) |
| FBgn0042111 | *CG18766* | 3R | 0.0088954 | (0.0043) | 1.9087111 | (<10^-4^) |
| FBgn0025678 | *CaBP1* | 2L | 0.0053115 | (0.0091) | 2.5415291 | (<10^-4^) |
| FBgn0025621 | *CG16989* | X | 0.0038405 | (0.0195) | 1.804998 | (<10^-4^) |
| FBgn0011274 | *Dif* | 2L | 0.0122409 | (0.0209) | 1.8945007 | (<10^-4^) |
| FBgn0050049 | *CG30049* | 2R | 0.0087456 | (0.0253) | 1.6336012 | (<10^-4^) |
| FBgn0028506 | *CG4455* | 2L | 0.0060465 | (0.0339) | 2.2240636 | (<10^-4^) |
| FBgn0038087 | *beat-Va* | 3R | 0.008767 | (0.0351) | 2.1084769 | (<10^-4^) |
| FBgn0038653 | *CG18208* | 3R | 0.0074815 | (0.0351) | 1.2692718 | (<10^-4^) |
| FBgn0020503 | *CLIP-190* | 2L | 0.0074815 | (0.0442) | 1.9601552 | (<10^-4^) |
| FBgn0266064 | *GlyS* | 3R | 0.0035662 | (<10^-4^) | 2.0604007 | (0.0183) |
| FBgn0261984 | *Ire1* | 3R | 0.0075888 | (0.0468) | 1.6305142 | (0.0183) |
| FBgn0038737 | *CG11447* |  |  |  |  |  |
| FBgn0016081 | *fry* | 3L | 0.0102732 | (<10^-4^) | 1.8448242 | (0.0218) |
| FBgn0036024 | *CG18180* |  |  |  |  |  |
| FBgn0036489 | *CG7011* | 3L | 0.0078538 | (0.0096) | 1.8361736 | (0.0218) |
| FBgn0036488 | *CG6878* |  |  |  |  |  |
| FBgn0036680 | *Cpr73D* | 3L | 0.0099152 | (0.0234) | 2.0042725 | (0.0218) |
| FBgn0261999 | *CG42817* | 2L | 0.0135467 | (<10^-4^) | 1.401196 | (0.0251) |
| FBgn0027094 | *Aats-ala* | 2L | 0.0057329 | (0.0003) | 2.1104726 | (0.0251) |
| FBgn0051928 | *CG31928* | 2L | 0.010294 | (0.0004) | 2.1035433 | (0.0251) |
| FBgn0053128 | *CG33128* |  |  |  |  |  |
| FBgn0261597 | *RpS26* | 2L | 0.0153538 | (0.0160) | 1.2594411 | (0.0251) |
| FBgn0051926 | *CG31926* | 2L | 0.007492 | (0.0235) | 2.1898388 | (0.0251) |
| FBgn0262024 | *CG42835* | 3R | 0.0128072 | (0.0043) | 1.9596318 | (0.0361) |
| FBgn0038509 | *CG14332* |  |  |  |  |  |
| FBgn0262869 | *Gfrl* | 3R | 0.0065385 | (0.0135) | 2.0865145 | (0.0361) |
| FBgn0015338 | *CG5861* | 2L | 0.0083128 | (0.0009) | 1.6930718 | (0.0491) |
| FBgn0011708 | *Syx5* |  |  |  |  |  |
| FBgn0002023 | *Lim3* | 2L | 0.0081011 | (0.0176) | 1.4929847 | (0.0491) |

**Table S3:** List of enriched GO terms and the genes grouped under this term for the European population in biological processes. The GO term shown in bold is the name of the group (the most significant term of the group).

| **GO Group** | **GO term** | | **Genes** | | | ***p*-value** | |
| --- | --- | --- | --- | --- | --- | --- | --- |
| **Group 1** | **cell morphogenesis involved in differentiation** | | ***Fhos, Ptpmeg, Sh, Src64B, acj6, chm, cnn, dnc, eIF-2beta, fry, haf, lea, lva, mys, nct, nub, pigs, plexA, rok, rut, vvl*** | | | **0.0002** | |
|  | cell morphogenesis involved in neuron differentiation | | *Ptpmeg, Sh, Src64B, acj6, chm, cnn, dnc, eIF-2beta, fry, haf, lea, lva, mys, nct, nub, plexA, rok, rut, vvl* | | | 0.0004 | |
|  | chemical synaptic transmission | | *Sh, VGlut, cac, dlg1, dnc, rab3-GAP, rut, stj, tomosyn* | | | 0.0021 | |
|  | axogenesis | | *Ptpmeg, Sh, Src64B, acj6, dnc, eIF-2beta, haf, lea, mys, plexA, rok, rut, vvl* | | | 0.0029 | |
|  | developmental growth | | *CG7246, Ptpmeg, Sh, Src64B, Ten-a, cac, dlg1, dnc, mys, rok, rut, stj, tlk* | | | 0.003 | |
|  | circadian behavior | | *5-HT1A, CkIIbeta, Ork1, Sh, cry, dlg1* | | | 0.0037 | |
|  | developmental growth involved in morphogenesis | | *Ptpmeg, Sh, dnc, mys, rok, rut* | | | 0.0038 | |
|  | regulation of circadian sleep/wake cycle, sleep | | *5-HT1A, Sh, cry* | | | 0.0073 | |
|  | axon extension | | *Ptpmeg, Sh, dnc, rut* | | | 0.0079 | |
|  | sleep | | *5-HT1A, CG42613, CG8329, Sh, cry, rut* | | | 0.0128 | |
|  | locomotor rhythm | | *CkIIbeta, Ork1, cry, dlg1* | | | 0.0165 | |
|  | mating behavior | | *5-HT1A, Pde1c, Sh, cac, dlg1* | | | 0.0181 | |
|  | regulation of behavior | | *5-HT1A, Sh, cry, rut* | | | 0.0226 | |
|  | detection of light stimulus | | *Sh, cac, cry, milt* | | | 0.0241 | |
|  | neuromuscular junction development | | *Src64B, Ten-a, cac, dlg1, rut, stj* | | | 0.0249 | |
|  | modulation of synaptic transmission | | *Sh, cac, rab3-GAP, tomosyn* | | | 0.0271 | |
| **GO Group** | | **GO term** | | **Genes** | ***p*-value** | |  |
| **Group 2** | | **regulation of stress fiber assembly** | | ***Fhos, mys, rok*** | **0.0013** | |  |
|  | | actomyosin structure organization | | *Fhos, Src64B, fliI, mys, rok* | 0.0022 | |  |
|  | | regulation of cell morphogenesis involved in differentiation | | *Fhos, fry, lva, plexA, rok, vvl* | 0.003 | |  |
|  | | actin filament bundle assembly | | *Fhos, Src64B, mys, rok* | 0.0031 | |  |
|  | | regulation of cellular component movement | | *Fhos, lea, mys, plexA* | 0.0077 | |  |
|  | | regulation of anatomical structure morphogenesis | | *Fhos, fry, kirre, lva, mys, nct, plexA, rok, tlk, vvl* | 0.0078 | |  |
|  | | regulation of cell morphogenesis | | *Fhos, fry, lva, mys, plexA, rok, tlk, vvl* | 0.0079 | |  |
|  | | regulation of locomotion | | *Fhos, lea, mys, plexA* | 0.0083 | |  |
|  | | regulation of cell migration | | *Fhos, lea, mys* | 0.0086 | |  |
|  | | regulation of cytoskeleton organization | | *Fhos, Src64B, cnn, mys, rok* | 0.0123 | |  |
|  | | regulation of dendrite morphogenesis | | *fry, lva, vvl* | 0.0158 | |  |
|  | | regulation of neuron differentiation | | *fry, lva, nct, plexA, rok, vvl* | 0.0165 | |  |
|  | | cell junction assembly | | *Src64B, kirre, rok* | 0.0306 | |  |
|  | | heart development | | *CHES-1-like, cora, lea, mys* | 0.0425 | |  |
| **Group 3** | | **central complex development** | | ***Ptpmeg, Ten-a, lea*** | **0.0013** | |  |
|  | | brain development | | *CG4853, CkIIbeta, Ptpmeg, Src64B, Ten-a, lea, vvl* | 0.0031 | |  |
|  | | mushroom body development | | *CG4853, CkIIbeta, Ptpmeg, Src64B, lea* | 0.0083 | |  |
|  | | neuron recognition | | *Ten-a, acj6, eIF-2beta, fry, lea, plexA* | 0.009 | |  |
|  | | synaptic target recognition | | *Ten-a, acj6, lea* | 0.0315 | |  |
| **Group 4** | | **organophosphate metabolic process** | | ***Ald, Ipk1, Pde1c, rut*** | **0.0315** | |  |
|  | | nucleotide metabolic process | | *Ald, Pde1c, rut* | 0.0417 | |  |
| **Group 5** | | **establishment of localization by movement along microtubule** | | ***klar, milt, rempA*** | **0.0289** | |  |
| **Group 6** | | **heart process** | | ***Ork1, cac, cora*** | **0.0306** | |  |
| **Group 7** | | **imaginal disc-derived wing hair organization** | | ***cora, fry, rok*** | **0.042** | |  |
| **Group 8** | | **positive regulation of developmental growth** | | ***CG7246, cac, dlg1, tlk*** | **0.0477** | |  |

**Table S4:** List of enriched GO terms and the genes grouped under this term for the European population for molecular function, cellular component and KEGG and Reactome pathways.

| **GO term** | **Genes** | ***p*-value** |
| --- | --- | --- |
| **Molecular Function** | | |
| cation channel activity | *Sh, cac, Ork1* | 0.0216 |
| transcription cofactor activity | *alien, chm, Gug, acj6* | 0.0287 |
| protein homodimerization activity | *Ork1, Hml, Trxr-1, lea, alien* | 0.0385 |
| **Cellular component** | | |
| apical part of cell | *rok, fry, Ser, cac, Cad96Ca, Megalin, dlg1* | 0.0160 |
| plasma membrane region | *Megalin, Cad96Ca, cac, Ser, mys, dlg1, Ten-a* | 0.0236 |
| microtubule | *klar, pigs, sub* | 0.0495 |
| **KEGG and Reactome** | | |
| ECM-receptor interaction | *Hml, CG3168, mys* | 0.0045 |
| neuronal system | *rut, dlg1, Vglut, elk, Ork1, Sh* | 0.0088 |
| G-alpha (s) signaling events | *dnc, Pde1c, rut* | 0.0108 |
| potassium channels | *elk, Ork1, Sh* | 0.0127 |
| cell-cell communication | *mys, kirre, Src64B* | 0.0138 |
| TGF-beta signaling pathway | *rok, sog, Pp2A-29B* | 0.0221 |
| EPH-Ephrin signaling | *Src64B, nct, rok* | 0.0353 |
| digestion of dietary lipid | *CG6847, CG4267, Yp1* | 0.0434 |
